# Characterization of transitional memory CD4+ and CD8+ T-cell mobilization during and after an acute bout of exercise

**DOI:** 10.3389/fspor.2023.1120454

**Published:** 2023-04-17

**Authors:** Rebekah M. Hunt, Mahmoud T. Elzayat, Melissa M. Markofski, Mitzi Laughlin, Emily C. LaVoy

**Affiliations:** ^1^ Laboratory of Integrated Physiology, Department of Health and Human Performance, University of Houston, Houston, TX, United States; ^2^ Houston Methodist Orthopedics and Sports Medicine, Houston Methodist, Houston, TX, United States

**Keywords:** lymphocytes, physical activity, leukocyte differentiation, exercise response, immunity

## Abstract

T-cell subsets, including naïve (NA), central memory (CM), transitional memory (TM), effector memory (EM), and RA + effector memory (EMRA), differ in phenotype and function. T-cells are mobilized by exercise, with differences in the magnitude of mobilization between subsets. However, the response of TM T-cells to exercise has not yet been described. Further, T-cells expressing the late differentiation marker CD57 are known to be highly responsive to exercise, but the relative response of CD57 + and CD57- within T-cell subsets is unknown. We therefore aimed to characterize the exercise-induced mobilization of TM T-cells, as well as to compare the exercise response of CD57 + and CD57- cells within T-cell subsets.

**Methods:**

Seventeen participants (7 female; aged 18–40 years) cycled 30 min at 80% of their estimated maximum heart rate. Venous blood obtained pre, post, and 1H post-exercise was analyzed by flow cytometry. CD45RA, CCR7, and CD28 expression within CD4 + and CD8+ T-cells identified NA, CM, TM, EM, and EMRA subsets. CD57 expression within EM, EMRA, and CD28+ T-cells was also quantified. The relative mobilization of each subset was compared by calculating fold change in cell concentration during (ingress, post/pre) and after exercise (egress,1H post/post). Cytomegalovirus (CMV) serostatus was determined by ELISA and was considered in models.

**Results:**

TM CD8+ T-cell concentration was greater post-exercise than pre-exercise (138.59 ± 56.42 cells/µl vs. 98.51 ± 39.68 cells/µl, *p* < 0.05), and the proportion of CD8 + with a TM phenotype was elevated 1H post-exercise (1H: 32.44 ± 10.38% vs. Pre: 30.15 ± 8.77%, *p* < 0.05). The relative mobilization during and after exercise of TM T-cells did not differ from NA and CM but was less than EM and EMRA subsets. Similar results were observed within CD4+ T-cells. CD57 + subsets of CD28+ T-cells and of EM and EMRA CD8+ T-cells exhibited a greater relative mobilization than CD57- subsets (all *p* < 0.05).

**Conclusion:**

These results indicate TM CD4 + and CD8+ T-cells are transiently mobilized into the blood with exercise, but not to as great of an extent as later differentiated EM and EMRA T-cells. Results also indicate CD57 identifies highly exercise responsive cells within CD8+ T-cell subsets.

## 1. Introduction

T-cell subsets differ in phenotype and function and can be identified by the combination of various proteins found on the cell surface. Mature naïve (NA) T-cells express CD45RA, CCR7, and CD28. Naive cells emerge from the thymus and become effector cells upon cognate antigen exposure; a portion of these cells then become subsets of memory T-cells (1). CD45RA-CCR7 + CD28 + central memory (CM) T-cells are earlier differentiated memory cells with longer telomeres than CD45RA-CCR7-CD28- effector memory (EM) T-cells; EM are later differentiated memory cells and have more immediate cytotoxic capacity (1, 2). Transitional memory (TM) T-cells, defined as CD45RA-CCR7-CD28+, have an intermediate differentiation status between CM and EM, with intermediate telomere length, proliferative potential, and cytotoxic function (3–5). Terminal effector memory cells that re-express the naïve marker (EMRA; defined as CD45RA + CCR7-CD28-) T-cells have the shortest telomere length of these T-cell subsets and exhibit low proliferation and low functional capacity (3, 5, 6). Many EMRA and EM T-cells also express the surface protein CD57, a marker associated with the lowest proliferative ability and increased susceptibility to apoptosis (4, 6). These CD28-CD57 + T-cells are frequently characterized as senescent, a designation suggesting DNA damage and the production of inflammatory proteins (7). Interestingly, CD28+ T-cells may also express CD57; these cells have increased effector functionality and proliferation ability compared to CD28-CD57+ (7).

Acute vigorous endurance exercise preferentially mobilizes later differentiated T-cells into peripheral blood (8–14). For example, using CD45RA in combination with CD27, CD62l, or CCR7, Campbell et al. (10) demonstrated a greater relative increase in EM and EMRA CD8+ T-cells in peripheral blood following 20 min of cycling exercise compared to NA and CM CD8+ T-cells. Similarly, Turner et al. (12) demonstrated a greater increase in late differentiated CD27-CD28- CD8+ T-cells following 60 min of vigorous running exercise than less differentiated cells. CD28-CD57+ T-cells are also significantly mobilized by exercise (13). This selective mobilization of later differentiated T-cells is due to the greater expression of type 2 beta-adrenergic receptors by highly differentiated T-cells, as epinephrine binding to these receptors promotes cell extravasation into the bloodstream (11, 15). After exercise ends, T-cells quickly egress from peripheral blood. Similar to the ingress during exercise, highly differentiated cells typically display a relatively larger egress out of peripheral blood than earlier differentiated cells (9–12, 14). The preferential mobilization of later differentiated cells means that individuals with a greater number of later differentiated cells display a larger cell mobilization response to exercise. This includes people infected with cytomegalovirus (CMV), a β-herpesvirus that leads to the accumulation of later differentiated T-cells (16).

Although the T-cell response to acute dynamic exercise has been fairly well characterized, there are still gaps in our knowledge. While TM T-cells are prevalent in healthy human subjects and are functionally distinct from CM and EM (3), their response to exercise has not been reported. Given the enhanced effector properties of TM relative to CM, the enhanced proliferative capacity of TM relative to EM, and the fact that TM T-cells comprise a substantial portion of the T-cells present in the blood (5), describing the mobilization of these cells by acute exercise is important to fully understanding the changes in T-cells that occur with exercise Further, while CD28-CD57+ T-cells are known to be mobilized by exercise (13), their relative mobilization compared to later differentiated subsets (EM, EMRA) lacking CD57 is not yet known. Finally, to our knowledge, there are no reports of the mobilization of CD28 + CD57+ T-cells with exercise. Given the enhanced functional and proliferative properties of these cells relative to CD28-CD57 + cells, understanding their degree of mobilization with exercise will provide further insight into the subsets of exercise-responsive T-cells. The practical importance of fully characterizing T-cell mobilization with exercise is highlighted by recent interest in the use of exercise as a tool to enhance immunotherapy (17–20).

The aims of our study were two-fold: first, to characterize the mobilization into and out of the peripheral blood of TM CD4 + and CD8+ T-cells relative to NA, CM, EM, and EMRA T-cells with exercise; and second, to compare the exercise response of CD57 + and CD57- cells within the T cell subsets. As latent infection with cytomegalovirus (CMV) has been associated with the heightened mobilization of T-cells into and out of the blood, we also considered the effects of CMV serostatus on the exercise-response of each T cell subset (16). We hypothesized that the relative mobilization of T-cells into the blood with exercise would increase with progressive differentiation; namely, NA < CM < TM < EM < EMRA. We also hypothesized that subsets expressing CD57 would show a greater mobilization compared to subsets lacking CD57, as later differentiated cells are highly mobilized by exercise and commonly express CD57.

## 2. Materials and methods

### 2.1. Experimental design and participants

This study was a secondary analysis of a larger investigation which consisted of 20 participants (10 female) between the ages of 18 and 40 years old who performed a cycling exercise in the Laboratory of Integrated Physiology at the University of Houston. All participants were recruited from Houston, TX. From this group, this secondary analysis examined 17 participants (seven female), from whom lymphocyte analyses were available. Participant characteristics are shown in Table 1. Participants were screened to ensure they were non-smokers (>10 years), exercised between one and six hours per week on average for the last six months, and met the American College of Sports Medicine criteria for participation in exercise (21). Exercise of any type was accepted for inclusion criteria and it should be noted that not all participants were trained cyclists. Participants were excluded if they reported a history of immune disease, or the regular use of medication known to affect the immune system. All participants gave written informed consent before participating in the study. This study was approved by the Institutional Review Board at the University of Houston (STUDY00000990).

**Table 1:** Participant characteristics and exercise performance measures. Values are presented as means ± SD.

Characteristics	All subjects (*n* = 17; 7 Female)
Age (years)	26.3 ± 4.5
Height (cm)	163.2 ± 13.2
Body Mass (kg)	72.7 ± 17.3
BMI (kg/m^2^)	24.1 ± 3.0
During Exercise:	
Mean Heart Rate (beats/min)	145 ± 4
Percent Maximum Heart rate^a^ (%)	80.6 ± 0.0
RPE	13 ± 2
Power (Watts)	137.6 ± 48.6
Relative Power Output (Watts/kg)	1.7 ± 0.4

^a^ Maximum heart rate estimated by (191.5-(0.007x(Age)^2^))(24). Mean heart rate (% max) estimated by max heart rate reached during exercise divided by age-predicted maximum heart rate.

^b^ Rating of perceived exertion, borg 6–20 scale.

For the current study, participants visited the laboratory twice. Participants with symptoms of upper-respiratory illness in the prior two weeks were rescheduled. Participant visits occurred between 6am and 10am. Participants were asked to refrain from strenuous exercise for 48 h, alcohol for 24 h, and food eight hours prior to their visit. During visit one, participants were screened and consented, and completed an incremental submaximal exercise test on a stationary bicycle. The results of this test were used to calculate the resistance corresponding to 80% of estimated maximal heart rate. During visit two, participants performed 30 min of bicycling exercise at this resistance.

### 2.2. Exercise bouts and blood collection

During visit one, participants completed a modified Åstrand test on a stationary bicycle (Velotron, RacerMate Inc., Seattle, Washington) (22). Briefly, participants cycled at a cadence of 70–90 revolutions per minute (RPM) at an initial resistance of 75–100 watts. Resistance was increased five to 10 watts every three minutes. Heart rate (HR; FT7 Polar, Polar United States) and rating of perceived exertion (RPE; Borg 6–20 scale) were recorded every minute (23). The test was completed when the participants surpassed 80% of their estimated maximum HR (calculated with the equation (191.5-(0.007x(Age)^2^)) (24), for a full three-minute interval. During the second visit, participants cycled for 30 min at the resistance found in visit one to correspond to 80% of their estimated maximal heart rate. Heart rate, RPE, and RPM were monitored throughout the trial, and resistance was altered as needed to maintain intensity within +/- 5 bpm of target heart rate. Participants were encouraged to drink water throughout the exercise session. Exercise performance measures are shown in Table 1.

Venous blood was collected from a vein in the antecubital space at three time points: pre-exercise, post-exercise, and 1 h (1H) post-exercise. Pre-exercise blood was collected following a 10 min rest prior to any cycling activity. Post-exercise blood was collected within two minutes of exercise cessation. Participants rested quietly until the 1H post-exercise sample was drawn. Blood was collected into 10 ml vacuum-sealed tubes treated with ethylene-diamine-tetra-acetic acid (EDTA; Becton, Dickinson, and Co., Franklin Lakes, NJ). An additional 10 ml blood sample was collected at rest using a serum collection tube (Vacutainer, BD). Blood was processed within three hours of collection.

### 2.3. Flow cytometry

**Figure 1:**
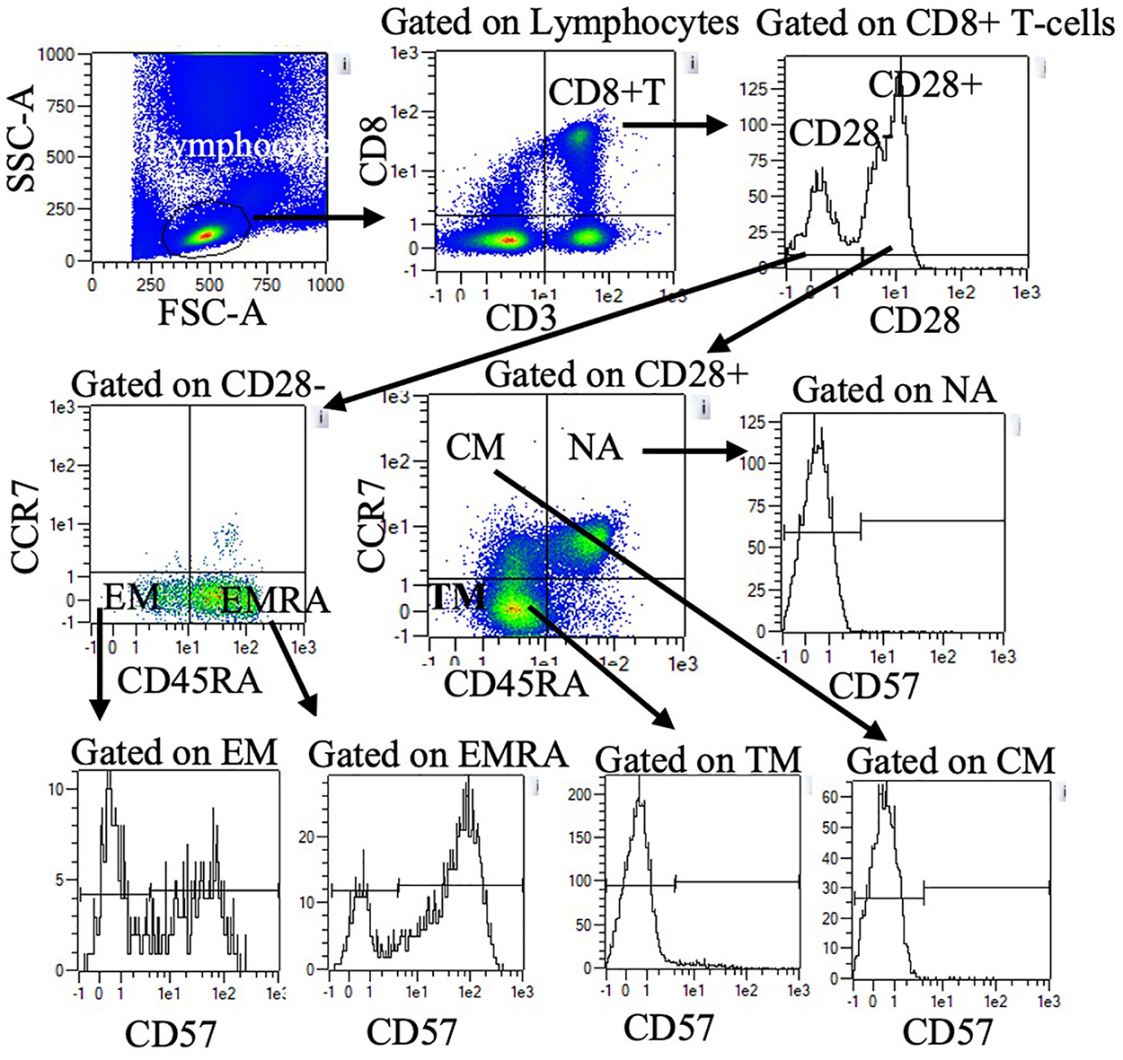
Representative flow cytometry data to illustrate gating strategy. Plots demonstrate sequential gating of lymphocytes, CD8+ T-cells, and CD28 + or CD28- CD8+ T-cells. Within CD28 + cells, NA, CM, and TM were identified by CD45RA and CCR7 expression. Within CD28- cells, EM, and EMRA were identified by CD45RA and CCR7 expression. CD57 + and CD57-cells within NA, CM, TM, EM, and EMRA were further quantified. A similar gating strategy was applied for CD4+ T-cells (data not shown). NA: naïve, CM: central memory, TM: transitional memory, EM: effector memory, EMRA: RA + effector memory.

Blood was treated with red blood cell lysis buffer (eBiosciences Inc., San Diego, CA) and stained with fluorescently tagged monoclonal antibodies against cell-surface antigens. Direct immunofluorescence assays were performed to identify proportions of T-cells using one of two panels. Panel one included Vioblue-anti-CD45RA (IgG2b, T6D11), Viogreen-anti-CD3 (IgG2ak, REA615), FITC- anti-CD4 (IgG2ak, VIT4) PE-anti-CCR7 (recombinant human (REA) IgG1, REA546), PE-Vio615-anti-KLRG1 (REA IgG1, REA261), PE-Vio770-anti-CD62l (IgG1κ, 145/15), APC-conjugated anti-CD28 (IgG1, REA612), and APC Vio770-conjugated anti-CD57 (IgMk, TB03). Panel two was identical to panel one except FITC-anti-CD8 (IgG2ak, BW135/80) was used in place of CD4. All antibodies were purchased from Milteny Biotech Inc. (Bergisch, Gladbach, Germany). Cells were incubated with 2 µl of the antibody in the dark at room temperature for 20 min, then washed and resuspended in 200 µl of phosphate buffered solution (Mediatech Inc., Manassas, VA). Cells were directly analyzed with MACSQuant analyzer flow cytometer (Milteny Biotec Inc., Bergisch, Gladbach, Germany). Compensation beads (Milteny Biotec Inc., Bergisch, Gladbach, Germany) were used to compensate for spectral overlap. Single color tubes were used to identify positive and negative staining by each antibody. Lymphocytes were gated electronically using MACSQuantify™ software. Within the lymphocyte gate, CD3 + CD4 + and CD3 + CD8+ T-cells were identified and further subdivided based on CCR7, CD45RA, CD28 and CD57. Figure 1 provides an example of the gating strategy. Table 2 summarizes CD4 and CD8 T cell subset phenotypes.

**Table 2:** Phenotypic identification of T cell subsets and functional properties.

Subset	Identification	Effector Properties	References
Naïve (NA)	CD45RA + CCR7 + CD28+	–	(3,4)
Central Memory (CM)	CD45RA-CCR7 + CD28+	–	(2–5)
Transitional Memory (TM)	CD45RA-CCR7-CD28+	+/-	(3–5)
Effector Memory (EM)	CD45RA-CCR7-CD28-	+	(2–5)
CD45RA + Effector Memory (EMRA)	CD45RA + CCR7-CD28-	+	(3–5)

### 2.4. Determination of viral serostatus

Serum samples were obtained by centrifugation and stored at −80°C until analysis. Viral serology (IgG) was determined for cytomegalovirus (CMV) in all subjects by ELISA following the manufacturer's instructions (BioCheck Inc., San Francisco, CA, United States). Results were read at 450 nm using a 96-well microplate reader (Molecular Devices, Sunnyvale, CA) and serum samples above the cut-off index were determined to be seropositive resulting in 12 seropositive subjects (6 female).

### 2.5. Statistical analysis

Prior research has indicated a medium to large effect of 30 min of cycling exercise on the concentration of peripheral blood leukocytes (14). Thus, *n* = 17 was expected to provide 80% power to detect differences in peripheral blood leukocytes due to exercise at *p* < 0.05.

Prior to analysis, data were screened for outliers (values >2.5SD from mean) and to assure all assumptions of normality and multicollinearity were met. Skewed data were transformed using log transformations where necessary. The concentrations of T cell subsets present in the blood at each time point were analyzed using random-intercepts maximum likelihood mixed models with a variance components covariance structure. Models included time (three time points) as the independent variable (16). Sex, CMV serostatus, time × sex, and time × CMV serostatus as independent variables were also considered for inclusion in models. Model selection was based on the minimization of Schwarz's Bayesian Criterion. Final models included time, CMV serostatus, and time × CMV serostatus. Significant results were assessed by pairwise comparison of estimated marginal means, adjusted for multiple comparisons by the method of Sidak. Effect sizes are reported as η^2^.

The fold change in T-cell subsets (concentration of cells post-exercise/concentration of cells pre-exercise; concentration of cells 1H post-exercise/concentration of cells post-exercise) were also analyzed using random-intercepts maximum likelihood mixed models with a variance components covariance structure. Models included subset (five subsets: NA, CM, TM, EM, EMRA; or two subsets: CD57+, CD57-) as independent variable. Sex, CMV serostatus, subset × sex, and subset × CMV serostatus as independent variables were also considered for inclusion in models; model selection was based on minimization of Schwarz's Bayesian Criterion. Final models included subset, CMV serostatus, and time × CMV serostatus as independent variables. Significant results were assessed by pairwise comparison of estimated marginal means, adjusted for multiple comparisons by the method of Sidak.

All analyses were completed using IBM SPSS Statistics for Windows, Version 28. *p* < 0.05 was accepted as significant.

## 3. Results

### 3.1. Exercise mobilizes TM T-cells into peripheral blood, independently of CMV serostatus

Exercise transiently mobilized CD4 + and CD8+ T-cells into peripheral blood (Table 3). The concentration of TM CD4 + and CD8+ T-cells in peripheral blood was significantly greater post-exercise than pre-exercise; values returned to baseline by 1H post-exercise. The concentration of NA, CM, EM, and EMRA CD4+ T-cells and CM, EM, and EMRA CD8+ T-cells was also significantly and transiently elevated with exercise.

**Table 3:** The concentration (cells/µl) of CD4 + and CD8+ T cell subsets present in peripheral blood Pre-, post-, and 1H post-exercise. Values are means ± S.D.; Main effects are F-statistic (*p*-value).

Cell subset:	Time			Main effect		Interaction effect
	Pre	Post	1H Post	Time	CMV	Time × CMV
CD4+ T Cells	627 ± 168	770 ± 181*,**	608 ± 120	**10.044 (<.001)**	1.435 (.248)	0.074 (.929)
NA	214 ± 88	253 ± 104*,**	189 ± 65	**8.979** (**<.001)**	0.217 (.647)	0.085 (.919)
CM	259 ± 102	305 ± 101*,**	253 ± 76	**11.454** (**<.001)**	**8.344** (**.011)**	1.234 (.305)
TM	134 ± 44	168 ± 50*	147 ± 56	**5.073** (**.012)**	0.461 (.507)	0.203 (.818)
EM	11 ± 11	25 ± 31*,**	10 ± 11	**38.857** (**<.001)**	**14.549** (**.002)**	0.681 (.513)
EMRA	4 ± 11	13 ± 35*,**	4 ± 11	**38.591** (**<.001)**	3.514 (.079)	0.760 (.476)
CD8+ T Cells	339 ± 120	512 ± 214*,**	302 ± 79	**15.438 (<.001)**	1.429 (.248)	0.800 (.458)
NA	106 ± 39	117 ± 41**	94 ± 31	**8.988** (**<.001)**	0.173 (.682)	0.775 (.469)
CM	24 ± 12	29 ± 14*,**	23 ± 10	**10.309** (**<.001)**	**5.812** (**.028)**	2.166 (.130)
TM	99 ± 40	139 ± 56*,**	98 ± 41	**14.755** (**<.001)**	0.611 (.445)	0.300 (.743)
EM	45 ± 35	92 ± 80*,**	33 ± 18	**50.788** (**<.001)**	3.220 (.091)	0.003 (.997)
EMRA	53 ± 46	118 ± 93*,**	43 ± 40	**56.112** (**<.001)**	4.277 (.054)	0.036 (.964)

Significance (*p* < .05) indicated by bold.

* (Differs from Pre).

** (Differs from 1H Post).

CMV serostatus impacted the concentration of CM CD4 + and CD8+ T-cells and EM CD4+ T-cells. CMV seropositive participants had a significantly lower concentration of CM CD4+ T-cells (F_(1,16)_ = 8.344, *p* = 0.011, η^2 ^= 0.343) and CM CD8+ T-cells (F_(1,17)_ = 5.812, *p* = 0.028, η^2 ^= 0.255). CMV seropositive participants had a greater concentration of EM CD4+ T-cells (F_(1,16)_ = 4.561, *p* = 0.049, η^2 ^= 0.222). CMV serostatus did not impact the concentration of TM CD4+ T-cells (F_(1,16)_ = 0.461, *p* = 0.507, η^2 ^= 0.028) nor TM CD8+ T-cells (F_(1,17)_ = 0.611, *p* = 0.445, η^2 ^= 0.035).

Exercise also significantly changed the proportions of CD4 + and CD8+ T-cell subsets in peripheral blood (Table 4). The proportion of TM CD4 + and CD8+ T-cells did not differ post-exercise relative to pre-exercise, but was significantly greater 1H post-exercise than both pre-exercise and post-exercise. The proportion of EM and EMRA CD4 + and CD8+ T-cells was significantly greater post-exercise and returned to baseline values 1H post-exercise. In contrast, the proportion of NA CD8+ T-cells was significantly decreased post-exercise and returned to baseline values 1H post-exercise.

**Table 4:** The proportions of CD4 + and CD8+ T-cell subsets in peripheral blood Pre-, post-, and 1H post-exercise. Values are means ± S.D.; Main and interaction effects are F-statistic (*p*-value).

Cell subset:	Time			Main effect		Interaction effect
	Pre	Post	1H Post	Time	CMV	Time × CMV
CD4+ T Cells						
NA	34 ± 9	32 ± 9**	31 ± 10*	**14.717** **(<.001)**	1.675 (.214)	**3.583** (**.039)**
CM	41 ± 10	40 ± 10	42 ± 10	3.208 (.054)	**5.961** (**.027)**	2.449 (.102)
TM	22 ± 7	22 ± 8**	24 ± 7*	**19.813** (**<.001)**	0.092 (.766)	2.772 (.078)
EM	2 ± 2	3 ± 4*^,^**	2 ± 2	**33.223** (**<.001)**	**16.632** (**<.001)**	1.124 (.338)
EMRA	1 ± 2	2 ± 4*^,^**	1 ± 2	**33.930** (**<.001)**	4.135 (.059)	1.112 (.341)
CD8+ T Cells						
NA	32 ± 6	24 ± 7*^,^**	31 ± 6	**31.212** (**<.001)**	0.738 (.402)	2.565 (.092)
CM	8 ± 4	6 ± 4*^,^**	8 ± 4	**26.964** (**<.001)**	**7.901** (**.012)**	1.462 (.246)
TM	30 ± 9	29 ± 11**	32 ± 10*	**6.831** (**.003)**	4.347 (.052)	0.224 (.800)
EM	12 ± 7	16 ± 9*^,^**	11 ± 6	**23.156** (**<.001)**	3.263 (.089)	0.477 (.625)
EMRA	14 ± 9	21 ± 12*^,^**	13 ± 10	**30.585** (**<.001)**	4.172 (.057)	0.913 (.411)

Significance (*p* < .05) indicated by bold.

* (Differs from Pre).

** (Differs from 1H Post).

CMV serostatus also impacted the proportions of CM CD4 + and CD8+ T-cells and EM CD4+ T-cells. CMV seropositive participants had a significantly lower proportion of CM CD4+ T-cells than seronegative participants (F(_1,16)_ = 5.961, *p* = 0.027, η^2 ^= 0.271), as well as a lower proportion of CM CD8+ T-cells (F_(1,17)_ = 7.901, *p* = 0.012, η^2 ^= 0.317). CMV serostatus did not impact the proportion of TM T-cells (F_(1,16)_ = 0.092, *p* = 0.766, η^2 ^= 0.006). Although not significant, there was a trend for a lower proportion of TM CD8+ T-cells amongst CMV seropositive (mean ± standard error: 37.171 ± 3.795% vs. 27.743 ± 2.450%; F_(1,17)_ = 4.347, *p* = 0.052, η^2 ^= 0.204).

A significant interaction effect between time and CMV serostatus was observed with the proportion of NA CD4 + T-cells. Post-hoc pairwise analyses indicate that among seropositive participants, the proportion of NA cells pre-exercise was significantly greater than post-exercise and 1H post-exercise (mean ± standard error: Pre: 35.7 ± 2.5% vs. Post: 33.5 ± 2.5% and 1H Post: 32.6 ± 2.5%; *p* < 0.01), whereas in CMV seronegative participants the proportion of NA cells pre-exercise was greater than 1H post exercise (Pre: 28.1 ± 4.3% vs. 1H Post: 25.7 ± 4.3%, *p* = 0.034) but not post-exercise (28.7 ± 4.3%, *p* = 0.894).

### 3.2. TM mobilization is similar to that of NA and CM T-cell subsets

**Figure 2:**
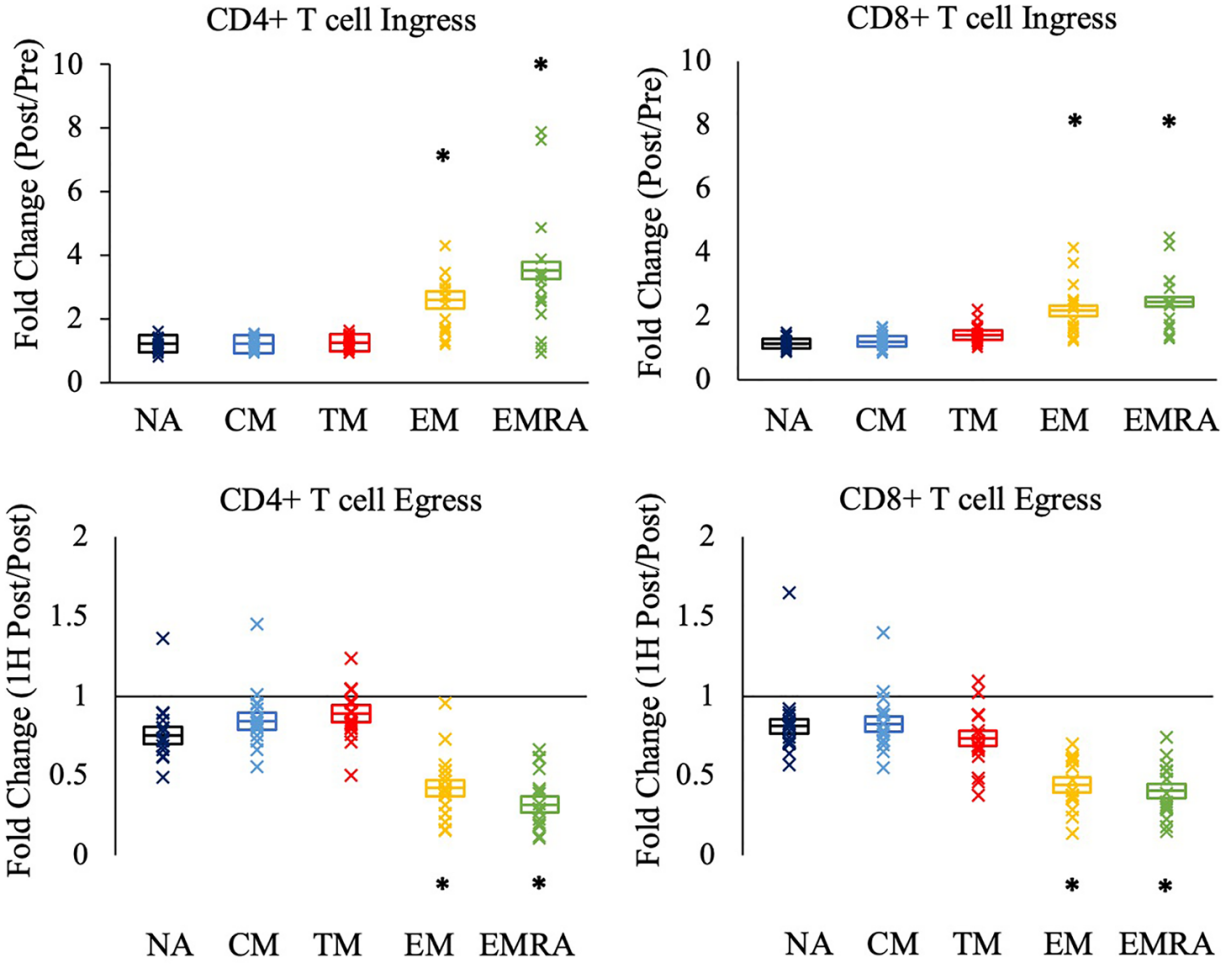
The relative mobilization of CD4 + and CD8+ T-cell subsets by an acute bout of exercise. Ingress (upper panels) reflects the fold change in the concentration of cells post-exercise relative to pre-exercise, and egress (lower panels) reflects the fold change 1H post-exercise relative to post-exercise. Data are displayed as estimated marginal means (line) and standard error (box). Individual data points from each participant are also indicated (x). Statistically significant differences (*p* < .05) in fold change from NA, CM, and TM is indicated by *. NA: naïve, CM: central memory, TM: transitional memory, EM: effector memory, EMRA: RA + effector memory.

We next asked if the relative mobilization into the blood post-exercise (fold change; ingress) and out of the blood 1H post-exercise (fold change; egress) differed between the CD4 + and CD8+ T cell subsets. Significant effects of subset were observed during ingress (CD4+ T-cells: F_(4,64)_ = 43.776, *p* < 0.001, η^2 ^= 0.732; CD8+ T-cells: F_(4, 68)_ = 44.505, *p* < 0.001, η^2 ^= 0.724) and egress (CD4+ T-cells: F_(4, 64)_ = 72.119, *p* < 0.001, η^2 ^= 0.818; CD8+ T-cells: F_(4, 68)_ = 50.190, *p* < 0.001, η^2 ^= 0.747). Post-hoc analyses revealed that among both CD4 + and CD8+ T-cells, the ingress and egress of TM CD4 + and CD8+ T-cells did not differ from NA and CM T-cells (Figure 2). Rather, EM and EMRA exhibited a larger fold change increase post-exercise and a larger fold change decrease 1H post-exercise compared to NA, CM, and TM.

No significant effect of CMV serostatus was observed for the ingress and egress of the T cell subsets (data not shown).

### 3.3. Magnitude of exercise response differs between subsets expressing CD57

**Figure 3:**
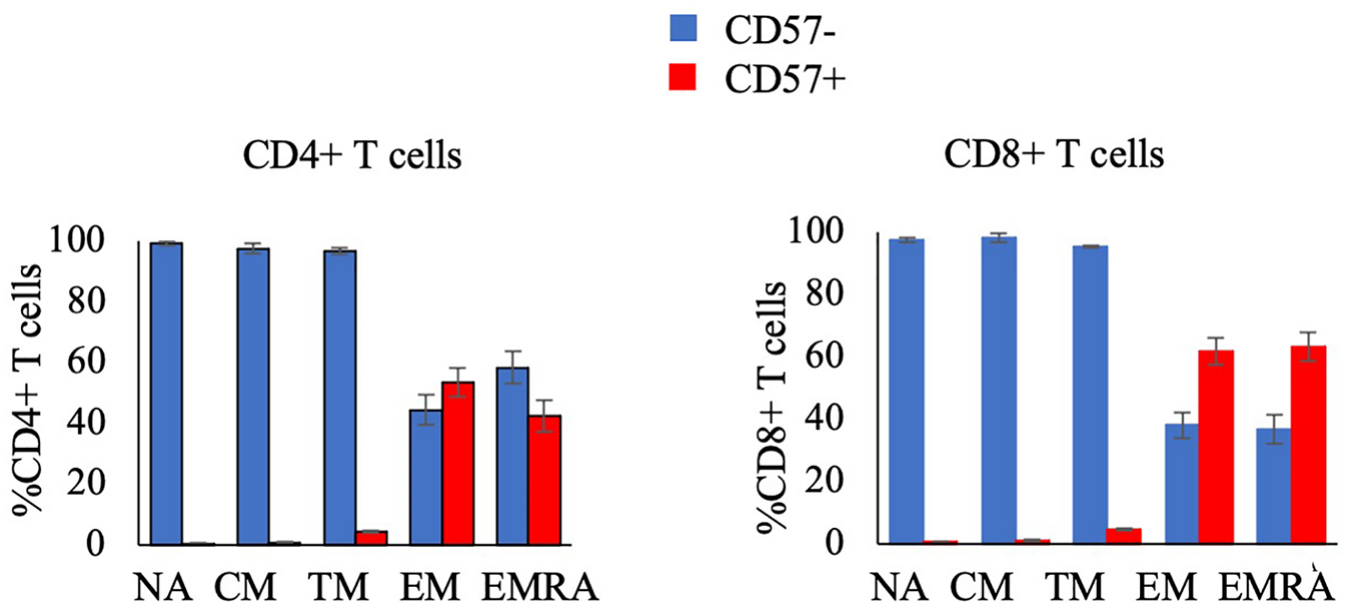
The proportion of CD4+ T-cell subsets (left panel) and CD8+ T-cell subsets (right panel) that are CD57- or CD57 + in resting blood. Data are displayed as mean and standard error (error bars).

The secondary aim of this study was to compare the response to exercise of CD57 + and CD57- cells within T cell subsets. The proportion and concentration of the NA, CM, and TM CD4 + and CD8+ T cell subsets that were CD57 + was very low (Figure 3). Therefore, we summed CD57 + and CD57- cells within the NA, CM, and TM subsets (each of which are CD28+) to analyze the relative mobilization of CD57 + and CD57- subsets of CD28+ T-cells.

**Figure 4:**
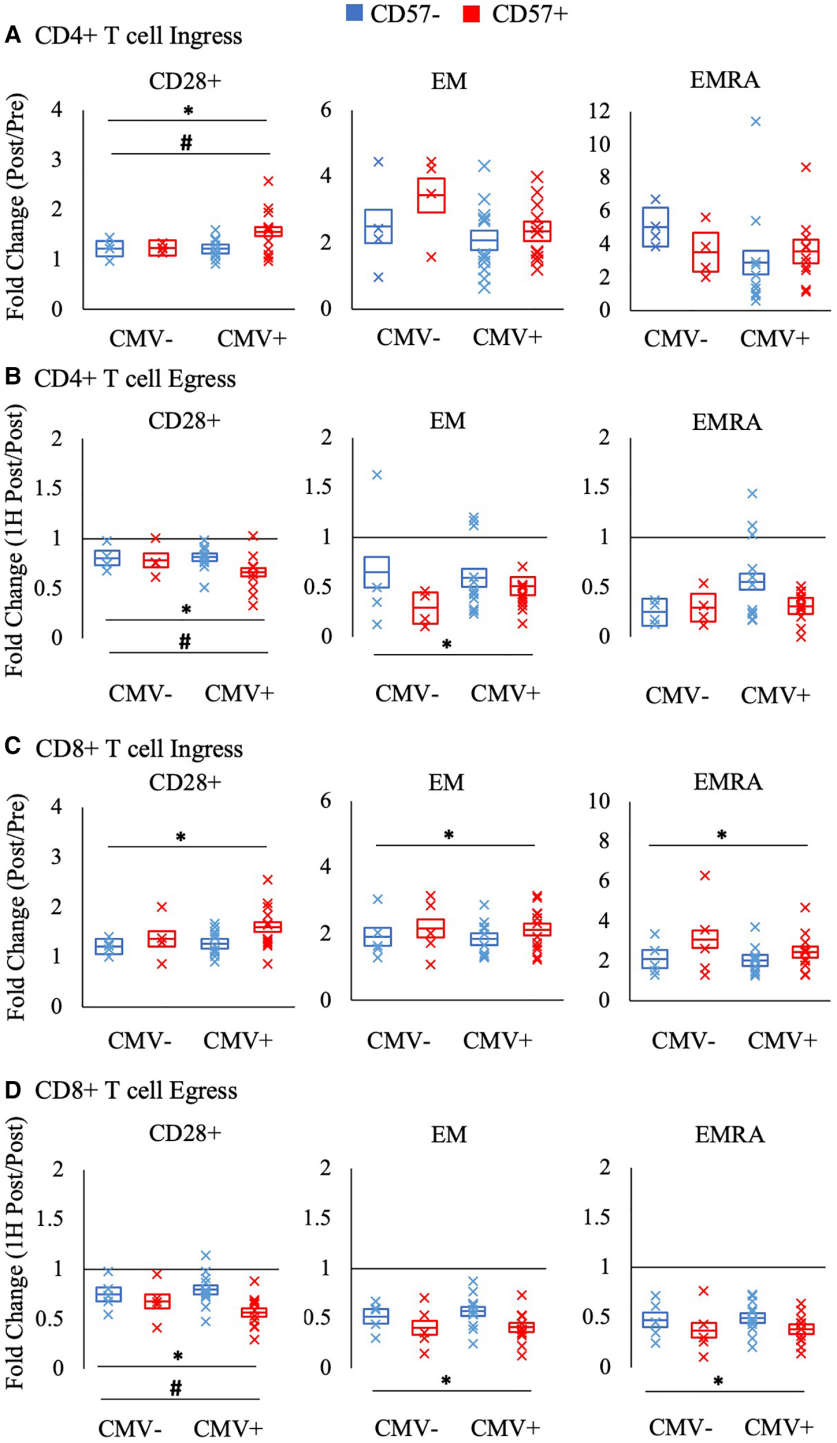
The relative mobilization of CD4 + and CD8+ T-cell subsets by CD57 expression. Ingress (panels A and C) reflects the fold change in the concentration of cells post-exercise relative to pre-exercise, and egress (panels B and D) reflects the fold change 1H post-exercise relative to post-exercise. The fold change in CD57 + and CD57- subsets of CD28+, EM, and EMRA CD4 + and CD8+ T-cells amongst CMV seronegative and seropositive participants is shown. Data are displayed as estimated marginal means (line) and standard error (box). Individual data points from each participant are also indicated (x). Significant main effect of CD57 expression indicated by *. Significant interaction effect between CD57 expression and CMV indicated by #. EM: effector memory, EMRA: RA + effector memory, CMV: cytomegalovirus.

Figure 4 displays the ingress and egress of CD4 + and CD8+ T cell subsets by CD57 expression. Among CD28+ CD4 + and CD8+ T-cells, CD57 + cells were mobilized significantly more than CD57- cells during ingress (CD4+ T-cells: F_(1,16)_ = 8.602, *p* = 0.010, η^2 ^= 0.350; CD8+ T-cells: F_(1, 17)_ = 7.879, *p* = 0.012, η^2 ^= 0.317) and egress (CD4+ T-cells: F_(1, 16)_ = 11.268, *p* = 0.004, η^2 ^= 0.413; CD8+ T-cells: F_(1, 17)_ = 21.027, *p* < 0.001, η^2 ^= 0.553). CD57+ EM CD8+ T-cells were mobilized significantly more than CD57- cells during ingress (F_(1, 17)_ = 14.471, *p* = 0.001, η^2 ^= 0.460) and CD57+ EM CD4 + and CD8+ T-cells displayed a greater egress than CD57- cells (CD4+ T-cells: F_(1, 16)_ = 9.430, *p* = 0.007, η^2 ^= 0.371; CD8+ T-cells: F_(1, 17)_ = 30.521, *p* < 0.001, η^2 ^= 0.642). CD57+ EMRA CD8+ T-cells also displayed a greater mobilization during ingress (F_(1, 17)_ = 16.087, *p* = 0.001, η^2 ^= 0.486) and egress (F_(1, 17)_ = 28.574, *p* < 0.001, η^2 ^= 0.627).

CMV serostatus significantly interacted with CD57 expression in the mobilization of CD28 + CD4+ T-cells (Figure 4). Post-hoc pairwise comparisons revealed that only CMV seropositive participants had a significant difference between CD57 + and CD57- cells in the ingress and egress of CD28+ CD4+ T-cells (interaction ingress: F_(1, 16)_ = 6.609; *p* = 0.021, η^2 ^= 0.292; egress: F_(1,16) _= 6.328; *p* = 0.023, η^2 ^= 0.283). A similar interaction effect was found for CD28 + CD8 + T cell egress (F_(1,17_) = 5.800; *p* = 0.028, η^2 ^= 0.254).

## 4. Discussion

The primary aim of this study was to characterize the mobilization of TM CD4 + and CD8+ T-cells by acute exercise. To the best of our knowledge, this subset of T cells, with distinct phenotype and function from CM and EM subsets, has not yet been examined in the context of exercise. We report that the concentration of TM CD4 + and CD8+ T-cells in peripheral blood is significantly increased post-exercise, and that this increase is transient, as TM T-cell concentration 1H post-exercise did not differ from resting values. In contrast, the proportion of CD4 + and CD8+ T-cells with a TM phenotype was significantly elevated 1H post-exercise compared to pre-exercise and post-exercise values. The relative mobilization of TM T-cells did not differ from NA and CM subsets, but was significantly less than EM and EMRA T-cell subsets. This indicates that TM are responsive to exercise, but not to the same extent as later differentiated subsets. The secondary aim of this study was to compare the mobilization of CD57 + and CD57- subsets of memory T-cells with acute exercise. We report that CD57 + subsets of EM and EMRA T-cells are mobilized to a greater extent than CD57- subsets. We further report that the unique subset of CD57 + CD28+ T-cells are mobilized to a greater extent than CD57-CD28 T-cells. Therefore, our results confirm and extend earlier findings that later differentiated T-cells are mobilized to a greater extent than earlier differentiated T-cells by acute exercise.

Our results are primarily in agreement with previous reports, as we found exercise transiently increases the concentration of T-cells in peripheral blood, particularly later differentiated subsets (25). This study expands upon earlier reports as we for the first time distinguish TM T-cells from EM T-cells. In prior work, the lack of a third surface marker prevented the identification of TM as distinct from EM (10, 12, 14). TM T-cells are an intermediate subset between CM and EM T-cells in terms of their telomere length, proliferation rates, expression of activation and co-stimulation receptors, and cytolytic molecule secretion (3, 4). For example, stimulated TM CD8+ T-cells produce higher levels of IFN-γ and granzyme A and B than CM T-cells, although not as much as EM T-cells. TM T-cells also express intermediate levels of CCR5 and CXCR3, suggesting the capacity to migrate to inflamed tissues (3). Given the intermediate phenotype and function, we also hypothesized that TM T-cells would show an intermediate level of mobilization with exercise, occurring between CM and EM T-cells. In contrast, TM did not differ from NA and CM, but was less than that of EM and EMRA subsets. The preferential mobilization of EM and EMRA has been attributed to the greater concentration of β-adrenergic receptors on the surface of these cells relative to NA and CM T-cells, allowing the enhanced response to exercise-induced increases in epinephrine (15). Although not measured in the current study, these findings suggest that TM T-cells do not express more β-adrenergic receptors than NA and CM T-cells. One-hour post-exercise, the proportion of TM CD4 + and CD8+ T-cells was significantly greater than pre-exercise. This was unique amongst the T-cell phenotypes examined, as NA, CM, EM, and EMRA cells had all returned to resting values or below. Indeed, we found TM T-cells comprised nearly one quarter of CD4 + T-cells and nearly one third of CD8+ T-cells 1H post-exercise. This indicates that the TM subset is an important component of the T-cell pool during the recovery from exercise, and ought to be considered and reported in future research.

This study also expands upon the existing literature characterizing the mobilization of CD57+ T-cells. Earlier work examining the mobilization of CD28-CD57 + or KLRG1 + CD57+ T-cells typically concluded that these cells were increased immediately post-exercise, with their concentration returning to resting values 1–3H post-exercise (25–28). In the current study, we examined CD57 expression within T -cell subsets defined by CD28, CCR7, and CD45RA. Not surprisingly, the expression of CD57 within the CD28 + subsets (NA, CM, and TM) was very low; thus we grouped these subtypes together for analyses (3, 4). Our results indicate that CD57 is a reliable marker of highly exercise-responsive CD8+ T-cells, even in the context of other phenotypic markers. While CD57 identified highly responsive CD28+ CD4+ T-cells, the mobilization of EM and EMRA CD4+ T-cells did not differ by CD57 expression. CD57 has been described as a marker of T-cell senescence, as it strongly associates with shortened telomeres and therefore reduced proliferative potential (4). However, CD57 expression also associates with high effector function, including expression of cytolytic molecules (29). Thus, the mobilization of CD57+ EM and EMRA CD8 + T-cells with exercise may indicate an increase in blood T-cells capable of rapidly responding to threats. Along these lines, post-exercise blood has been shown to contain a greater concentration of functional, virus-specific T-cells (8, 30). Further, while CD57 expression often coincides with the loss of CD28, a unique population of CD28 + CD57+ T-cells has been described (7). These double-positive T-cells appear to be activated, and have greater expression of regulatory molecules including IL-10, PD-1, and CTLA-4 compared to CD28 + CD57- and CD28-CD57+ T-cells. Thus, the increase in these double positive T-cells with exercise observed in the current study may indicate greater regulation of the immune system. However, the proportion of these cells was low in comparison to CD57+ EM and EMRA T-cells.

Due to persistent antigen exposure, latent infection with CMV can drive the accumulation of CD28-CD57+ T-cells (7, 31, 32). Given that these cells are preferentially mobilized with exercise, it follows that CMV seropositive individuals demonstrate a greater mobilization of CD8+ T-cells and T-cell subsets (12, 14, 16, 33, 34). We therefore considered CMV serostatus in the current study; however, although CMV seropositive participants had an elevated concentration and proportion of EM CD4+ T-cells, no significant differences due to CMV serostatus were noted among EM CD8+ T-cells or EMRA T-cells. This lack of a CMV effect on cell concentration likely explains the lack of effect on relative mobilization, as we found no differences between CMV seropositive and seronegative participants in the magnitude of mobilization of the EM and EMRA T-cells. There was a non-significant trend for CMV seropositive participants to have a lower proportion of TM CD8+ T-cells overall relative to CMV seronegative participants. This, alongside the trend for CMV seropositive participants to have a greater proportion of EMRA CD8+ T-cells and slight trend for greater proportion of EM CD8+ T-cells suggests that CMV infection leads to the accumulation of the later differentiated effector memory cells rather than and at the expense of the TM CD8+ T-cell compartment. It is possible that our trending data may have reached the threshold for significance if we had a larger sample size. However, earlier studies reporting an effect of CMV on T-cell mobilization with exercise employed a similar sample size. We speculate that the differences between the current study and earlier work regarding the influence of CMV may be due to our inclusion of both males and females in the current study; a feature that was lacking in the earlier work that recruited only male participants. Sex differences exist in the epinephrine response to aerobic exercise as well as in leukocyte β-adrenergic receptor expression and thus males and females may display differences in the degree of mobilization of T-cell subsets with exercise (35, 36). However, sex was not included as a factor in the current analyses and so this remains speculative. We did consider the addition of sex as an independent variable when building the statistical models, but its addition did not improve model fit and so was not included. We note that this study was not designed to consider sex differences and did not collect data regarding oral contraceptive pill use nor stage of menstrual cycle (35, 36).

In addition to the limitations of not considering oral contraceptive pill use or menstrual cycle phase in female participants, an additional limitation is that we did not measure cell function (i.e., cytokine expression and cytotoxicity) or telomere length. Thus, we cannot conclude that changes in T-cell subset concentration and/or proportion lead to differences in immune function with exercise. Another limitation is that the number of CMV seropositive and seronegative participants was unequal. This study was a secondary analysis of a separate study that did not include CMV serostatus as a variable of interest, and so we were unable to recruit equal numbers of CMV positive and CMV negative participants.

In conclusion, TM CD4 + and CD8+ T-cells are responsive to acute aerobic exercise. The concentration of TM T-cells is transiently increased immediately post-exercise, whereas the proportion of TM T-cells is elevated 1H post-exercise. TM T-cells display a relative mobilization similar to other CD28 + subsets, NA and CM. Results also indicate CD57 identifies highly exercise responsive cells within CD8+ T-cell subsets, as well as within CD28 + CD4 + T-cells. Collectively, these data expand our understanding of the mobilization of T-cells by exercise.

## Data Availability

The raw data supporting the conclusions of this article will be made available by the authors, without undue reservation.
